# Women in Biosensors Science

**DOI:** 10.3390/bioengineering10050603

**Published:** 2023-05-18

**Authors:** Rossana E. Madrid

**Affiliations:** Media and Interfaces Laboratory (LAMEIN), Bioengineering Department, Faculty of Exact Sciences and Technology (FACET), National University of Tucumán, Superior Biological Research Institute (INSIBIO), CONICET, Av. Independencia 1800, San Miguel de Tucuman 4000, Argentina; rmadrid@herrera.unt.edu.ar; Tel.: +54-381-436-4120

From the first glucose biosensor from Updike and Hicks (1968), there was an explosion of research in biosensors for detecting a wide range of analytes. Biosensors are preferred over laboratory equipment, due to their high specificity and sensitivity, wide linearity range, fast response, robustness, simple and cheap fabrication, and flexibility of use [1].

Since the 1980s, the development of biosensors has grown enormously, starting with electrochemical first-generation biosensors, then second-generation ones with the use of mediators, until the last decade, when nanotechnology enabled the development of third-generation biosensors enabled by direct electron-transfer. In turn, the development of biosensors for a wide range of applications, from health to the environment, precision agriculture, pharmacology, and defense and security, among others, has allowed the ubiquity of these devices. The use of nanotechnology has, in turn, allowed the development of highly sensitive devices, some of which are able to detect analyte concentrations as low as picogram or femtogram. Other advantages of using nanotechnology are direct electron transfer, preferential orientation of biomolecules on device surfaces, and miniaturization. The largest market is in the medical area, particularly biosensors for blood glucose detection for diabetics. Currently, the global trend is to apply biosensors for personalized medicine [2]. However, for the development of these customized devices, the use of new materials that allow a much more efficient and biomimetic abio–bio interface of the transducer was key. This has been possible thanks to polymers of biological origin. In particular, the use of hydrogels in recent years has gained popularity and has become a mainstay in biological and biomedical applications, since it allows the immobilization of the bioreceptor in a highly hydrated and porous medium, which retains the native conformation of the biorecognition elements, and allows a greater number of these immobilized elements. This material is a three-dimensional cross-linked polymer of highly hydratable monomers which, when exposed to water, swell to balance the monomer’s tendency to dissolve and the shrinkage force of the polymeric network, and whose hydration is highly controllable. However, its most important characteristic is its cellular and tissue biocompatibility and therefore its high potential for synthetic molecular engineering [3].

In the near future, the inclusion of biosensors in microfluidic systems for the manufacture of lab-on-a-chip devices, the development of wearable devices, embedded systems, systems with wireless communication, or artificial intelligence, are undoubtedly such important advances that will make biosensors common devices in people’s lives. This necessarily requires that the research and developments go to a later stage of transfer and commercialization. This will allow for the early diagnosis of diseases, improvement in the quality of crops, more adequate development of drugs, food safety, etc., and will improve society’s quality of life [3]. Additionally, this is the purpose of all research in any area.

On the other hand, contributions on biosensors of biological signals are also included in this Special Issue, in particular, sensor technology for the determination of physical signals instead of chemical ones. Sensors in the hand allow the reflection of, for example, tasks of robotic hands. Through interdisciplinary studies of neuroscience, interesting contributions can be made in the area of biomedical robotic assistive devices.

At this point, it is worth noting the contributions of women and men around the world to make a difference. Undoubtedly, women in science, and especially women pursuing STEM careers can bring their full potential in this regard. However, all the people whose main inspiration is to make a valuable contribution and to transform society for a better future, must be highlighted and valued, because science is essential for society.

Very important contributions were published in this Special Issue, including four original articles and one review. It is worth noting the article of Buonanno et al., who made an interesting contribution to the frequency-response enhancement of microwave resonant sensors in continuous non-invasive blood glucose monitoring [4]. This contribution is particularly important, since as we said, the glucose biosensor market is very widespread and broad. Moreover, as is known, non-invasive detection is required by patients suffering from diabetes, since it avoids having to prick themselves several times a day. The authors carried out an in-depth analysis of three processing methodologies, with comparisons, advantages, and disadvantages of each one, first evaluating synthetic signals, specifically generated ad hoc. After that, they validated the measurements with experimental data from glucose and water solutions. They evaluated three algorithms: the single-step algorithm, the iterative algorithm (both based on pairs of direct inverse Fourier transformations), and the super-resolution MUltiple Signal Classification (MUSIC) algorithm. By using a Monte Carlo analysis, they found that all the evaluated methods were satisfactory to accurately and precisely estimate the resonant frequency and, therefore, to determine the blood glucose concentration, but the best one was the one-step algorithm, which provides the best performance.

Another interesting contribution to this Special Issue is the work of Liu et al. which presents an interdisciplinary study of systems neuroscience and cognitive behavioral science to monitor grip forces during hand and finger movements, which can be applied to learning in robotic surgeries [5]. Based on previous studies, the authors analyze individual grip force data in robotic hand control. They compared the grip forces of both hands (dominant and non-dominant) with handheld wireless sensors, comparing the forces of a highly competent expert and a complete novice for manual robot control. Using a neural network model, the differences between expert and novice performance with respect to grip force variability are evaluated. Measurements on the dominant hand showed finger synergies that reflect the skill of the robotic task. The results obtained by the authors would allow real-time monitoring of grip strength, which would allow assessment of learning evolution or identification of individual proficiency levels. These studies will contribute to improving new types of robotic surgeries, such as endoscopic and laparoscopic ones.

The work of De León-Hernández provides interesting data processing that can be used for transduction in the characterization and sensing of non-adherent cell suspensions [6]. The work proposes two approaches to evaluate the electrical double-layer capacity in the impedance measurement of a suspension of particles or non-adherent cells (K-562 cells and leukocytes). The first approach corresponds to a procedure for calculating the normalized electrical impedance spectrum which the authors named the Disperse Medium Index (DMi). The second approach is modeling by means of an equivalent electrical circuit using a parameter that unifies the double-layer phenomena, which they called effective capacitance (Cef).

The authors first found that the DMi is a function of particle concentration and size by analyzing a colloidal suspension of polymer particles of different diameters. Similar results were found for suspensions of the K-562 cells and leukocytes. On the other hand, the effective capacitance presents a well-distinguishable curve depending on the concentration for each type of cell, so it would represent a more reliable parameter. The same presents better results to analyze the sample size, although it requires an optimization algorithm to determine the values of the components of the electrical circuit. With this approach, the authors presented a simple method to characterize a suspension of non-adherent cells in a culture medium.

Turning to the biosensors themselves, Chiou et al. present an important work where they develop an electrochemical biosensor for the detection of specific exosomal miRNAs (exomiR) in the urine of prostate cancer patients [7]. The exomiR biosensor developed allows for the prediction of tumor progression since, according to the exosomes detected and their levels, it is possible to know the stage of the tumor, whether there is lymphatic metastasis, or whether the tumor was adequately removed.

The biosensor is based on the use of a screen-printed carbon electrode functionalized with a two-tipped probe made of single-stranded DNA complementary (ssDNA) to exomiR-21 or exomiR-451. The transduction principle is electrochemical, and the current of the redox response of the HRP enzyme used to amplify the signal is measured. Two electrochemical methods (cyclic voltammetry and chronoamperometry) were used to detect the sample. The authors were able to compare clinical urine samples between patients with prostate cancer, benign prostatic hypertrophy, and normal subjects, with significant results, allowing the use of the biosensor for the accurate detection of urinary miRNA. The results were verified by the consistency of serum PSA detection, the standard method for prostate cancer detection.

Finally, a review from Wang et al. presents the recent advances in CRISPR/Cas-Based biosensors for protein detection [8]. The authors present a review of the application of CRISPR/Cas biosensors as a sensitive and specific tool for protein analysis since studies on CRISPR applications for protein detection are still relatively scarce. For this purpose, they analyzed the works presenting CRISPR/Cas-based biosensors for protein detection considering their mechanism of action in three aspects: antibody-assisted CRISPR/Cas-based protein detection, aptamer-assisted CRISPR/Cas-based protein detection, and miscellaneous CRISPR/Cas-based methods for protein detection. ELISA is the gold standard method for protein detection. However, it is still not sensitive enough for the rapid detection of ultra-low concentrations of protein biomarkers.

From the papers reviewed, the authors concluded that CRISPR/Cas biosensors combined with antibodies have greatly improved protein LODs and extended the detection range. Most of the CRISPR-based biosensors for protein detection analyzed use aptamers as recognition elements. On the other hand, the authors state that with the rapid development of SELEX technology, more and more aptamers for proteins will be discovered, which will greatly expand the applications of this type of biosensor for protein detection.

The authors found that, to date, most reported methods rely on antibodies and aptamers, with some strategies in conjunction with other methods. However, these methods generally have the disadvantages of multi-step detection, ease of contamination, the need for specialized technicians, and reduced reliability in real samples. In addition, they still have long detection times, which is not compatible with the needs of clinical use. All this can be improved by combining it with other technologies such as automation, the incorporation of microfluidic and paper-based microfluidic technology, as well as bioinformatics, which would surely improve the sensitivity and specificity of biosensing through gRNA/crRNA design and target triggers. In turn, the combination of multiple Cas enzymes with different functions would allow multiple assays to be run simultaneously.

Finally, considering the rapid development of technology, the increasing research on CRISPR/Cas systems, and the discovery of new CRISPR/Cas systems, the authors foresee that CRISPR/Cas technology will become one of the main protein detection tools, which will facilitate their rapid development and application in various fields, starting with the medical and environmental fields, and many more.

The contributions to this Special Issue allow readers to explore the vast field of biosensors, discovering new and interesting applications that make use of new technologies such as CRISPR/Cas or exosomal biomarkers. New developments in this field will surely allow new areas of application to be covered, and that will inspire ideas for future research in the area, which will surely revolutionize the field of biosensing.
